# High-Resolution Mass Spectrometry for Identification, Quantification, and Risk Assessment of 40 PFAS Migrating from Microwave Popcorn Bags

**DOI:** 10.3390/molecules30091989

**Published:** 2025-04-29

**Authors:** Jen-Yi Hsu, Huei-Jie Jiang, Chih-Wei Chang, Yuan-Chih Chen, Pao-Chi Liao

**Affiliations:** 1 Department of Environmental and Occupational Health, College of Medicine, National Cheng Kung University, Tainan 704, Taiwan; superkawai0714@gmail.com (J.-Y.H.); s78094029@gs.ncku.edu.tw (C.-W.C.); tp6m0654@gmail.com (Y.-C.C.); 2 Department of Food Safety/Hygiene and Risk Management, College of Medicine, National Cheng Kung University, Tainan 701, Taiwan; j89121275@gmail.com

**Keywords:** perfluoroalkyl and polyfluoroalkyl substances, microwave popcorn bags, migration test, risk assessment, high-resolution mass spectrometry

## Abstract

Perfluoroalkyl and polyfluoroalkyl substances (PFASs) are widely utilized in food contact materials (FCMs) due to their water- and oil-repellent properties, yet their potential migration into food raises significant health concerns. This study employs high-resolution mass spectrometry (HRMS) to quantify the migration of 40 PFAS from microwave popcorn bags and assess the associated health risks. HRMS offers high mass accuracy and resolution, enabling precise detection of a broad spectrum of PFASs, including those with low migration levels. Migration experiments were conducted using 10% ethanol and 50% ethanol as food simulants at 70 °C for 2 h. The results indicate that when risk assessment is based solely on the European Food Safety Authority’s (EFSA) tolerable weekly intake (TWI) for four PFAS, hazard ratio (HR) values range from 0.01 to 0.8, suggesting minimal risk. However, when all PFAS are converted into perfluorooctanoic acid equivalents (PEQs) and compared against the U.S. Environmental Protection Agency’s (EPA’s) reference dose (RfD), HR values range from 0.3 to 142.3, indicating a significantly elevated health risk. These findings emphasize the necessity of comprehensive risk assessments incorporating the cumulative effects of all PFAS to better understand potential human exposure and inform regulatory policies.

## 1. Introduction

Perfluoroalkyl and polyfluoroalkyl substances (PFASs) are a group of synthetic compounds characterized by their hydrophobic, oleophobic, and surface tension-reducing properties. Since their introduction in the 1950s, PFASs have been extensively utilized in a wide range of commercial and industrial applications, including food contact materials (FCMs), cookware, textiles, and aqueous film-forming foams [1]. The carbon–fluorine bond, recognized as the strongest known covalent bond (450 kJ/mol) [2], imparts exceptional thermal stability to PFAS, rendering them highly resistant to environmental degradation. Consequently, PFAS has a tendency to bioaccumulate in organisms. Notable members of the PFASs group include perfluorooctanoic acid (PFOA), perfluorononanoic acid (PFNA), perfluorooctanesulfonic acid (PFOS), and perfluorohexanesulfonic acid (PFHxS), among others. Growing evidence indicates the toxicity of PFASs, linking exposure to liver damage [3], thyroid dysfunction [4], metabolic disorders [5], reproductive issues [6], and cancer [7].

In order to prevent the persistence and pollution of PFASs in the environment, international regulatory measures were implemented, with the Stockholm Convention issuing restrictions on the use of PFOS and its salts in 2009 [8]. The Convention subsequently called for the elimination of PFOA, PFHxS, and related compounds in 2019 [9] and 2022 [10]. The European Food Safety Authority (EFSA) established a tolerable weekly intake (TWI) for the sum of PFOA, PFNA, PFHxS, and PFOS in 2020, setting the limit at 4.4 ng/kg body weight (bw)/week (0.63 ng/kg bw/day) [11]. In 2023, the International Agency for Research on Cancer (IARC) classified PFOA as a Group 1 carcinogen and PFOS as a Group 2B substance [12]. In 2024, the U.S. Environmental Protection Agency (EPA) also established an overall noncancer reference dose (RfD) for PFOA of 0.03 ng/kg bw/day based on immune, developmental, and cardiovascular outcomes [13]. These environmental, health, and regulatory concerns have increased public awareness of PFAS-related risks.

PFAS contamination has been detected in human serum, with studies between 2007 and 2018 identifying PFOS as the predominant compound, followed by PFOA, PFHxS, and PFNA in adult serum samples [11,14,15]. In Taiwan, a study conducted in 2013 measured PFOS and PFOA concentrations in 59 serum samples, revealing PFOS concentrations ranging from 3.45 to 25.65 ng/mL, and PFOA concentrations between 1.55 and 7.69 ng/mL. The data also indicated an age-related increase in PFOS concentrations [16]. FCMs represent a primary exposure route for humans to PFAS, with migration of these substances occurring when FCMs come into contact with food or during food heating processes [17]. The European Commission Recommendation 2019/794 [18] emphasizes investigating PFAS in paper and board-based materials, including those used for fast food packaging, takeaway containers, bakery products, and microwave popcorn bags, in order to evaluate the potential migration of chemicals from FCMs into food. Numerous studies have focused on the extraction and quantification of PFAS in microwave popcorn bags, revealing detectable concentrations of these substances [2,19,20,21,22,23,24,25,26,27]. It has been observed that microwave popcorn bags often contain higher levels of PFAS compared to other FCMs [22,23,25,28]. However, fewer studies have examined the migration of PFAS from these bags into food. Existing research has demonstrated that only a limited number of PFAS compounds migrate from microwave popcorn bags [29,30,31]. A recent study by Chen et al. (2022) performed PFAS extraction and quantification in microwave popcorn bags in Taiwan [25], but the migration of PFAS from these bags into food has not been comprehensively assessed. Additionally, although high-resolution mass spectrometry (HRMS) analysis offers high mass accuracy and resolution [32], only a limited number of studies have employed HRMS for the quantitative analysis of PFAS migration from FCMs [33]. Therefore, the present study aims to monitor the migration of 40 different PFAS compounds from microwave popcorn bags into food using HRMS and to conduct exposure and risk assessments to evaluate the potential health risks posed to consumers.

## 2. Results

In the present study, migration tests were conducted in accordance with European Union (EU) regulations, utilizing food simulant A (10% ethanol) and food simulant D1 (50% ethanol) at an incubation temperature of 70 °C for a duration of 2 h. The findings demonstrated that microwave popcorn bags exhibited significantly higher migration concentrations of PFAS compared with other FCM samples reported in previous studies. Specifically, the migration concentrations ranged from 5.2 ± 0.4 ng/g (6.9 ± 0.7 ng/dm^2^) to 274.0 ± 7.0 ng/g (340.9 ± 5.6 ng/dm^2^). Additionally, following the EFSA approach for estimating the weekly intake, and considering that PFHxS and PFOS were not monitored in the migration tests, only the sum of PFOA and PFNA was included in the calculation. The hazard ratio (HR) values ranged from 0.01 to 0.8. However, many of the risks associated with the monitored PFAS remain unassessed. Therefore, following the RPF (relative potency factor) approach [34], other PFAS were converted into perfluorooctanoic acid equivalents (PEQ), and the HR was calculated relative to the PFOA reference dose (RfD) established by the US EPA [13]. The HR results ranged from 0.3 to 142.3. With the exception of sample P1, the HR values for the other four samples were all greater than 1. This suggests that, when considering all PFAS, microwave popcorn bags may pose a potential health risk.

### 2.1. Calibration Curve

Table 1 presents the calibration parameters, including the coefficient of correlation (R), limit of quantification (LOQ), linear range, and relative standard error (RSE) for the 40 compounds analyzed. As indicated in Table 1, the correlation coefficient (R) for all 40 compounds is ≥0.99, which satisfies the criteria outlined in the Food Chemistry Testing guidelines established by the Taiwan Food and Drug Administration (TFDA). Furthermore, the RSE for all compounds is within 20%, in accordance with the requirements of US EPA Method 1633 [35]. The formula for calculating the RSE is as follows: $RSE=100\times \sqrt{\sum_{i=1}^{n}\frac{{\left[\frac{xi^{\prime }-xi}{xi}\right]}^{2}}{n-p}}$. x_i_ is the theoretical concentration of each calibration standard, x_i_^′^ is the measured concentration of each calibration standard, n is the number of concentration points in the calibration curve, and p is the type of curve (2 = linear).

However, to ensure the condition during the analysis, both 2xLOQ and the midpoint concentration (C4) standards were used in the analysis process along with the samples, and both accuracy and precision were well below the criteria set by US EPA Method 1633 [35].

### 2.2. Migration Test Results

Table 2 demonstrates the migration of various PFAS compounds across five microwave popcorn bag samples. The migration concentration range for each sample is as follows: for P1, 5.2 ± 0.4 ng/g (6.9 ± 0.7 ng/dm^2^) to 166.6 ± 10.1 ng/g (215.9 ± 9.1 ng/dm^2^); for P2, 8.6 ± 0.5 ng/g (11.4 ± 0.4 ng/dm^2^) to 118.3 ± 6.5 ng/g (156.5 ± 2.5 ng/dm^2^); for P3, 9.7 ± 0.6 ng/g (13.1 ± 0.7 ng/dm^2^) to 166.3 ± 8.1 ng/g (220.1 ± 2.9 ng/dm^2^); for P4, 26.5 ± 1.1 ng/g (32.9 ± 0.2 ng/dm^2^) to 274.0 ± 7.0 ng/g (340.9 ± 5.6 ng/dm^2^); and for P5, 14.9 ± 0.3 ng/g (18.1 ± 0.3 ng/dm^2^) to 165.6 ± 11.9 ng/g (199.5 ± 13.2 ng/dm^2^). This indicates that the P4 sample exhibited the highest migration concentration. It can also be observed from Table 2 that PFHxA, PFHpA, and PFOA migrated in both food simulant A (10% ethanol) and food simulant D1 (50% ethanol) in all five samples. Furthermore, in samples P2, P3, P4, and P5, PFNA and PFDA were also found to migrate in both simulants. In addition, PFUnA and PFDoA were found to migrate in food simulant D1 (50% ethanol) in samples P2, P3, and P4, whereas no migration of these compounds was observed in food simulant A (10% ethanol). Table 2 also highlights that the concentration changes in samples P1, P2, and P5 in the two food simulants (10% ethanol and 50% ethanol) show little variation, indicating that their migration behavior is relatively consistent. However, the concentration of sample P4 in 50% ethanol is significantly higher than in 10% ethanol, while the concentration of sample P3 is higher in 10% ethanol than in 50% ethanol. In this study, to minimize analytical variability, migration tests were conducted in triplicate and analyzed on the same day. Therefore, the observed variations are likely attributable to differences in the composition of the coating formulations, which may influence their interactions with the respective food simulants.

### 2.3. Risk Assessment

In this study, two risk assessment approaches were employed. In the EFSA approach, the TWI threshold encompasses four PFAS compounds: PFOA, PFNA, PFHxS, and PFOS. However, since PFHxS and PFOS were not detected in the migration tests, the estimated weekly intake (EWI) calculation was based solely on the sum of PFOA and PFNA. However, the risks of five other monitored PFAS were not evaluated. Therefore, in the RPF approach, the RPF was utilized to convert the remaining PFAS into PEQ, enabling the calculation of the HR relative to the PFOA RfD established by the US EPA [13]. Table 3 presents the PEQ values for each PFAS after conversion. This table demonstrates that the compound with the highest PEQ in the P1 sample is PFOA, with concentrations of 5.2 ng/g in food simulant A (10% ethanol) and 5.8 ng/g in food simulant D1 (50% ethanol). In the other samples, PFDA exhibited the highest PEQ. In food simulant A (10% ethanol), the PEQ of PFDA for the P2 sample ranged from 124.7 ng/g to 311.8 ng/g; for P3 it ranged from 133.8 ng/g to 334.6 ng/g; for P4 it ranged from 116.3 ng/g to 290.8 ng/g; and for P5 it ranged from 127.2 ng/g to 318.0 ng/g. In food simulant D1 (50% ethanol), the PEQ of PFDA for the P2 sample ranged from 255.4 ng/g to 638.5 ng/g; for P3 it ranged from 212.9 ng/g to 532.3 ng/g; for P4 it ranged from 575.2 ng/g to 1437.9 ng/g; and for P5 it ranged from 144.6 ng/g to 361.6 ng/g. The second-highest PEQ value was observed for PFNA. Furthermore, Table 3 also indicates that the P4 sample, in both food simulant A (10% ethanol) and food simulant D1 (50% ethanol), exhibited the highest total PEQ compared to all other samples. The total PEQ for P4 in food simulant A (10% ethanol) ranged from 693.7 ng/g to 907.3 ng/g, while in food simulant D1 (50% ethanol), it ranged from 2057.1 ng/g to 2987.5 ng/g.

The dietary exposure and risk assessment are presented in Table 4. In this table, the HR calculated using the EFSA approach ranges from 0.01 to 0.8, indicating that when not all PFASs are considered, none of the five microwave popcorn bag samples pose a health risk. However, when applying the RPF approach and converting all monitored PFAS into PEQ, the HR ranges from 0.3 to 142.3. With the exception of sample P1, where the HR ranges from 0.3 to 1.2, the HR for the other four samples is significantly greater than 1, indicating that when all PFASs are considered, all five microwave popcorn bag samples pose a health risk. Furthermore, Table 4 also shows that contact with food simulant D1 (50% ethanol) results in higher HR values compared to contact with food simulant A (10% ethanol), indicating that the health risks associated with exposure to lipophilic foods are greater than those from exposure to hydrophilic foods.

## 3. Discussion

Previous studies have indicated that PFAS may contribute to background contamination, which can originate from solvents, glassware, and laboratory equipment [26]. To address this potential source of contamination, method blanks were utilized to confirm the absence of such background contamination in the present study. In order to minimize the risk of PFAS contamination, all equipment was thoroughly cleaned before and after use following the protocols outlined in US EPA Method 1633 [35], employing water, methanol, and methanolic ammonium hydroxide. The results of the method blanks demonstrated no detectable contamination from PFHxA, PFHpA, PFOA, PFNA, PFDA, PFUnA, or PFDoA, thereby ensuring that the migration concentrations observed in the study were not influenced by background contamination.

Regarding the calibration curve, the RSE for all 40 compounds analyzed was maintained within the specification outlined in US EPA Method 1633 [35], where the RSE needs to be less than 20%. In this study, the RSE values for the compounds ranged from 0.4% to 7.9%. The compound with the lowest RSE was PFBA, while PFTrDA exhibited the highest RSE. For the PFAS compounds migrating from microwave popcorn bags, the RSE values were as follows: PFHxA (2.2%), PFHpA (0.8%), PFOA (2.8%), PFNA (2.2%), PFDA (2.8%), PFUnA (1.9%), and PFDoA (2.7%), indicating a high degree of accuracy in the calibration curve.

This study monitored the migration of 40 distinct PFAS compounds from microwave popcorn bags to food, surpassing the scope of previous studies, which typically focused on a limited number of PFAS compounds. Earlier investigations primarily concentrated on other FCMs, such as paper bags, paper plates, baking paper, and muffin cups, with few studies addressing the migration and conducting risk assessments for microwave popcorn bags. For example, T. H. Begley et al. [29] reported that the migration of PFOA from microwave popcorn bag samples was below 3 ng/dm^2^. Kevin M. Stroski et al. [30] did not monitor migration from microwave popcorn bags, and Agnieszka A. Niklas et al. [31] only monitored the migration of 6:2 FTOH, with a concentration of 294 ng/g. In contrast, our study monitored the migration of seven PFAS compounds, including PFHxA, PFHpA, PFOA, PFNA, PFDA, PFUnA, and PFDoA. As presented in Table 2, the minimum migration concentration in this study was 5.2 ± 0.4 ng/g (6.9 ± 0.7 ng/dm^2^), which was higher than the maximum migration concentration reported by T.H. Begley et al. [29] The highest migration concentration observed in this study was PFOA, with a concentration of 274.0 ± 7.0 ng/g (340.9 ± 5.6 ng/dm^2^), which represents the highest concentration among all compounds analyzed. This value is similar to the results reported by Agnieszka A. Niklas et al. [31].

In terms of risk assessment, Michaela Lerch et al. [36] reported that when they calculated only the sum of PFOA and PFNA, the adult CEDI from paper plates and muffin cups ranged from 0.06 to 0.12 ng/kg bw/day. However, when using the RPF approach, the adult CEDI ranged from 0.35 to 1.87 ng/kg bw/day. In comparison, when we calculated only the sum of PFOA and PFNA, the CEDI for adults ranged from 0.01 to 0.5 ng/kg bw/day, while using the RPF approach, the adult CEDI ranged from 0.01 to 4.3 ng/kg bw/day. These findings indicate that the migration of PFAS from microwave popcorn bags is similar to and greater than that from paper plates and muffin cups. Additionally, Yelena Sapozhnikova et al. [33] reported that the EWI of PFAS migrating from FCMs, such as cake paper, salami paper, and cookie-coated paper, ranged from 0.0006 to 1.12 ng/kg bw/week (0–0.1 ng/kg bw/day). Kevin M. Stroski et al. [37] found that the EWI from plastic food storage bags was 2.12 ng/kg bw/week (0.3 ng/kg bw/day). Both of these studies used the RPF approach to convert PFAS into PEQ and calculate the CEDI. For comparison with our study, the CEDI results were used to calculate the HR values based on the US EPA RfD for PFOA (0.03 ng/kg bw/day), resulting in HR values of 0–3.3 and 10, respectively. However, in our study, except for Sample P1, for which the HR ranged from 0.3 to 1.2, the HR values for the other four microwave popcorn bag samples ranged from 21.9 to 142.3. These results suggest that the migration of PFAS from microwave popcorn bags may pose a higher health risk compared to paper bags and plastic food storage bags.

## 4. Materials and Methods

### 4.1. Chemicals and Reagents

All the reagents used in this study were of LC grade and LC-MS grade. Methanol was purchased from Merck (Darmstadt, Germany), while acetonitrile and ethanol were purchased from J.T. Baker (Phillipsburg, NJ, USA). Ammonium acetate was purchased from Sigma-Aldrich (St. Louis, MO, USA). The native standards of PFAS and their isotopically labeled internal standards were purchased from Wellington Laboratories (Guelph, ON, Canada). The 40-compound native standard mixture solution prepared with methanol includes the following compounds and their respective concentrations: PFHxA, PFHpA, PFOA, PFNA, PFDA, PFUnA, PFDoA, PFTrDA, PFTeDA, PFBS, PFPeS, PFHxS, PFHpS, PFOS, PFNS, PFDS, PFDoS, PFOSA, NEtFOSA, NMeFOSA, NMeFOSAA, and NEtFOSAA at a concentration of 250 ng/mL; PFPeA, PFMPA, PFMBA, NFDHA, and PFEESA at 500 ng/mL; PFBA, 4:2FTS, 6:2FTS, 8:2FTS, HFPO-DA, ADONA, 9Cl-PF3ONS, and 11Cl-PF3OUdS at 1000 ng/mL; 3:3FTCA at 1250 ng/mL; NEtFOSE and NMeFOSE at 2500 ng/mL; and 5:3FTCA and 7:3FTCA at 6250 ng/mL. The 24-compound internal standard mixture solution prepared with methanol includes the following compounds at the following concentrations: M9PFNA, M6PFDA, M7PFUnA, MPFDoA, and M2PFTeDA at 250 ng/mL; M5PFHxA, M4PFHpA, M8PFOA, M3PFBS, M3PFHxS, M8PFOS, M8PFOSA, d-N-MeFOSA, and d-N-EtFOSA at 500 ng/mL; M5PFPeA, M2-4:2FTS, M2-6:2FTS, M2-8:2FTS, d3-N-MeFOSAA, and d5-N-EtFOSAA at 1000 ng/mL; M4PFBA and M3HFPO-DA at 2000 ng/mL; and d7-N-MeFOSE and d9-N-EtFOSE at 5000 ng/mL.

### 4.2. Sample Collection and Preparation

Five microwave popcorn paper bag samples were purchased from online stores in Taiwan. To ensure the integrity of the analysis, these samples did not come into contact with food. The samples were then cut into 1 cm × 1 cm pieces, and each piece was weighed for subsequent calculations.

### 4.3. Migration Tests

The selection of food simulants and migration conditions was based on the guidelines outlined in EU regulations for plastic materials and articles intended for food contact [38]. Given the nature of microwave popcorn bags, which come into contact with both corn and butter, two food simulants were chosen: a 10% ethanol solution (food simulant A) and a 50% ethanol solution (food simulant D1). Food simulant A was selected to simulate hydrophilic characteristics, while food simulant D1 was chosen to represent the lipophilic properties of oil-in-water emulsions, which are relevant to the composition of microwave popcorn. The migration time and temperature were determined according to standardized testing protocols specified in the EU regulations. Given that the boiling point of ethanol is 78 °C, migration test number OM3 was selected, with migration conditions set at 70 °C for a duration of 2 h. First, the weighed 1 cm^2^ microwave popcorn bag samples were placed into a 5 mL Eppendorf tube. Each tube was then filled with 4 mL of either a 50:50 (*v*:*v*) ethanol–water solution (food simulant D1) or a 10:90 (*v*:*v*) ethanol:water solution (food simulant A). Experiments were conducted in triplicate for each condition, and method blanks were included to control for potential contamination. The method blanks were prepared using two types of food simulants and underwent the same experimental process as the samples. The tubes were subsequently placed in a water bath and incubated at 70 °C for 2 h. After incubation, 4 mL of the supernatant from each tube was transferred to a 15 mL polypropylene tube under nitrogen gas. The extract was evaporated to dryness under a stream of nitrogen gas, then reconstituted to a final volume of 200 μL with methanol, followed by centrifugation at 20,000× *g* for 10 min. Finally, 58.8 μL of the supernatant was transferred to polypropylene autosampler vials, and 1.2 μL of the internal standard mixture was added to each sample for analysis using liquid chromatography–high-resolution mass spectrometry (LC-HRMS) (Thermo Fisher Scientific, Bremen, Germany).

### 4.4. LC-HRMS Analysis

Liquid chromatographic separation (LC) was conducted using an Ultimate 3000 system (Thermo Fisher Scientific, Bremen, Germany), coupled with an Orbitrap Q Exactive Plus hybrid mass spectrometer (Thermo Fisher Scientific, Bremen, Germany). Chromatographic separation was achieved on a Phenomenex Luna Omega polar C18 column (100 × 2.1 mm, 1.6 μm, Phenomenex, Torrance, CA, USA). The mobile phase consisted of two components: (A) 2 mM ammonium acetate in a 95:5 water–acetonitrile mixture and (B) acetonitrile. The gradient conditions for the separation were as follows: from 0 to 1 min, 2% B; from 1 to 12 min, a linear increase from 2% to 95% B; from 12 to 14 min, 95% B; from 14 to 15 min, a linear decrease from 95% to 2% B; and from 15 to 16 min, 2% B. The separation process was performed at a flow rate of 250 μL/min and maintained at a column temperature of 45 °C. The injection volume was set to 5 μL. Electrospray ionization (ESI) was performed in negative ion mode. For HRMS analysis, full scan mode was employed, with a scanning range of 100 to 1000 *m*/*z* and a resolution of 70,000 for the MS. The relevant information for the analyzed compounds, including retention time (RT), detected ions, and precursor ions, is detailed in Table 5. Data acquisition and subsequent processing were conducted using Skyline 22.2.0.351 (Seattle, WA, USA).

### 4.5. Calibration Curve

In the quantitative analysis, the LOQ, calibration curves, and RSE were established in accordance with US EPA Method 1633 [35]. The calibration curve was constructed by performing least-squares linear regression with a weighting factor of 1/χ, utilizing seven concentration points. For the quantitative analysis of the analytes, the peak area of each analyte standard was normalized by the corresponding isotopically labeled internal standard. The quantitative criteria for analysis included a signal-to-noise (S/N) ratio greater than 10, mass accuracy within ±5 ppm, and RT for target analytes with their corresponding isotopically labeled internal standards within ±0.1 min. For target analytes without isotopically labeled internal standards, quantification was performed using surrogate internal standards, and the RT was required to be within ±0.4 min of the surrogate. The corresponding internal standards are listed in Table 5. The calibration curve met the criteria of RSE ≤ 20% and a R ≥ 0.99. These criteria were adapted from US EPA Method 1633 [35] and the Food Chemistry Testing guidelines of the TFDA [39]. However, in order to ensure the condition of the instruments during the analysis, the instrument was calibrated with both 2xLOQ and the midpoint concentration (C4) standards every 10 injections, and accuracy and precision were based on the criteria of US EPA Method 1633 [35].

### 4.6. Risk Assessment

Dietary exposure was calculated in accordance with the guidance provided by the U.S. Food and Drug Administration (US FDA) [40]. The CEDI calculation requires the incorporation of consumption factors (CF) and food-type distribution factors (f_T_) as outlined by the US FDA. CF represents the fraction of the daily diet expected to be in contact with specific packaging materials, while f_T_ indicates the fraction of FCMs expected to interact with aqueous, acidic, alcoholic, and fatty foods. This study used two approaches for risk assessment. The EFSA has evaluated human exposure risks associated with PFAS in food and recommended a combined maximum TWI for PFOA, PFNA, PFHxS, and PFOS of 4.4 ng/kg bw/week (0.63 ng/kg bw/day). Therefore, this study used the EFSA approach to assess the risk of these four compounds. Additionally, to account for other monitored PFAS, the RPF approach was used to convert the monitored PFAS concentrations into PEQ [34], which were then used for comparison with the EPA’s recommended overall noncancer RfD of 0.03 ng/kg bw/day [13].

Finally, the HR was calculated [41], where the HR is the ratio of the CEDI to the RfD. An HR value greater than 1 suggests a higher potential health risk. The equations are as follows:

(1) PEQ = Concentration of each PFAS in food simulant (ng/g) × RPF.
(2) CEDI = Total amount of PFAS (ng/g) × CF × f_T_ × Amount consumed per day (g)/70 (kg).
(3) HR = CEDI/RfD.

## 5. Conclusions

This study employed HRMS to monitor the migration of 40 PFASs from five microwave popcorn bags and successfully detected seven PFASs, including PFHxA, PFHpA, PFOA, PFNA, PFDA, PFUnA, and PFDoA. When the EFSA approach was applied, the calculated HR ranged from 0.01 to 0.8, suggesting minimal health risks. However, when the U.S. EPA’s RfD was used and the RPF approach was applied to convert PFAS concentrations into PEQ, the HR ranged from 0.3 to 142.3. With the exception of sample P1, the HR values of the remaining samples significantly exceeded 1, indicating potential health concerns under high-exposure assumptions. Furthermore, the HR values in food simulant D1 (50% ethanol) were consistently higher than those in simulant A (10% ethanol), and two additional PFAS compounds (PFUnA and PFDoA) were detected only in simulant D1. These results indicate the higher migration potential of PFAS in lipophilic environments. This study represents the first PFASs migration risk assessment of microwave popcorn bags conducted in Taiwan. As the EFSA provides TWI values for only four PFAS compounds (PFOA, PFNA, PFHxS, and PFOS), our findings suggest that the exclusion of other PFAS may underestimate potential health risks. Importantly, the elevated HR values calculated via the RPF approach emphasize the possible health implications of cumulative PFAS exposure. However, it is essential to emphasize that the potential health risks associated with daily consumption of a single bag of microwave popcorn are based on high intake assumptions and the use of the RPF approach as reported in the literature, a method which currently lacks widely accepted regulatory guidelines. Therefore, these results should be interpreted with caution. These findings highlight the inadequacy of current regulatory values for a comprehensive risk assessment of PFAS and emphasize the necessity of calculating cumulative exposure to all PFAS. However, previous studies have indicated that the interactions among different PFAS compounds may depend on the composition of the mixture. For instance, mixtures involving PFOS often exhibit synergistic effects, while those with PFOA may show either antagonistic or synergistic effects [42]. Nevertheless, these compound interactions remain largely unclear [43]. Therefore, future studies should broaden the scope of PFAS monitoring, further elucidate their toxicological effects, and validate the feasibility of the RPF approach to enhance the comprehensiveness of risk assessments and support the development of more inclusive PFAS regulatory standards.

## Figures and Tables

**Table 1:** Calibration parameters, R, LOQ, linear range, RSE, accuracy and precision for the compounds analyzed.

Compounds	Abbreviation	R	Slope	LOQ (ng/mL)	Linear Range (ng/mL)	RSE (%)	Accuracy (%)		Precision (%)	
							2 × LOQ	C4	2 × LOQ	C4
Perfluorobutanoic acid	PFBA	0.999	2.60 × 10^−2^	0.8	0.8–250	0.4	101	101	1	1
Perfluoropentanoic acid	PFPeA	0.999	5.49 × 10^−2^	0.4	0.4–125	1.0	102	102	1	1
Perfluorohexanoic acid	PFHxA	0.999	1.05 × 10^−1^	0.2	0.2–62.5	2.2	106	101	0	0
Perfluoroheptanoic acid	PFHpA	0.999	1.03 × 10^−1^	0.2	0.2–62.5	0.8	99	101	0	0
Perfluorooctanoic acid	PFOA	0.999	7.28 × 10^−2^	0.2	0.2–62.5	2.8	96	101	0	0
Perfluorononanoic acid	PFNA	0.999	1.51 × 10^−1^	0.2	0.2–62.5	2.2	100	100	1	1
Perfluorodecanoic acid	PFDA	0.999	2.43 × 10^−1^	0.2	0.2–62.5	2.8	102	100	0	0
Perfluoroundecanoic acid	PFUnA	0.999	2.45 × 10^−1^	0.2	0.2–62.5	1.9	103	100	1	1
Perfluorododecanoic acid	PFDoA	0.999	2.33 × 10^−1^	0.2	0.2–62.5	2.7	102	100	0	0
Perfluorotridecanoic acid	PFTrDA	0.999	2.83 × 10^−1^	0.2	0.2–62.5	7.9	105	103	2	2
Perfluorotetradecanoic acid	PFTeDA	0.999	2.17 × 10^−1^	0.2	0.2–62.5	1.8	108	102	1	1
Perfluorobutanesulfonic acid	PFBS	0.999	1.12 × 10^−1^	0.2	0.2–62.5	1.3	101	101	1	1
Perfluoropentanesulfonic acid	PFPeS	0.999	1.38 × 10^−1^	0.2	0.2–62.5	0.9	102	100	0	0
Perfluorohexanesulfonic acid	PFHxS	0.999	9.57 × 10^−2^	0.2	0.2–62.5	1.2	102	100	0	0
Perfluoroheptanesulfonic acid	PFHpS	0.999	1.55 × 10^−1^	0.2	0.2–62.5	1.9	103	100	1	1
Perfluorooctanesulfonic acid	PFOS	0.999	9.41 × 10^−2^	0.2	0.2–62.5	4.8	101	100	1	1
Perfluorononanesulfonic acid	PFNS	0.999	9.65 × 10^−2^	0.2	0.2–62.5	1.5	102	100	0	0
Perfluorodecanesulfonic acid	PFDS	0.999	6.99 × 10^−2^	0.2	0.2–62.5	5.1	102	101	1	1
Perfluorododecanesulfonic acid	PFDoS	0.999	4.25 × 10^−2^	0.2	0.2–62.5	2.1	105	101	1	1
1*H*,1*H*, 2*H*, 2*H*-Perfluorohexane sulfonic acid	4:2FTS	0.999	7.94 × 10^−2^	0.8	0.8–250	1.5	104	101	1	1
1*H*,1*H*, 2*H*, 2*H*-Perfluorooctane sulfonic acid	6:2FTS	0.999	2.64 × 10^−2^	0.8	0.8–250	1.4	101	100	1	1
1*H*,1*H*, 2*H*, 2*H*-Perfluorodecane sulfonic acid	8:2FTS	0.999	2.91 × 10^−2^	0.8	0.8–250	1.9	102	101	0	0
Perfluorooctanesulfonamide	PFOSA	0.999	8.29 × 10^−2^	0.2	0.2–62.5	1.0	100	101	1	1
N-methyl perfluorooctanesulfonamide	NMeFOSA	0.999	8.81 × 10^−2^	0.2	0.2–62.5	1.9	102	100	0	0
N-ethyl perfluorooctanesulfonamide	NEtFOSA	0.999	4.55 × 10^−2^	0.2	0.2–62.5	4.3	101	101	1	1
N-methyl perfluorooctanesulfonamidoacetic acid	NMeFOSAA	0.999	4.31 × 10^−2^	0.2	0.2–62.5	4.1	99	101	1	1
N-ethyl perfluorooctanesulfonamidoacetic acid	NEtFOSAA	0.999	5.83 × 10^−2^	0.2	0.2–62.5	1.1	100	103	2	2
N-methyl perfluorooctanesulfonamidoethanol	NMeFOSE	0.999	6.10 × 10^−2^	2	2–625	2.1	101	105	3	3
N-ethyl perfluorooctanesulfonamidoethanol	NEtFOSE	0.999	6.21 × 10^−2^	2	2–625	3.3	101	102	3	3
Hexafluoropropylene oxide dimer acid	HFPO-DA	0.999	4.85 × 10^−1^	0.8	0.8–250	1.4	102	101	1	1
4,8-Dioxa-3*H*-perfluorononanoic acid	ADONA	0.999	4.95 × 10^0^	0.8	0.8–250	2.5	102	101	1	1
Perfluoro-3-methoxypropanoic acid	PFMPA	0.999	4.30 × 10^−2^	0.4	0.4–125	1.2	99	101	0	0
Perfluoro-4-methoxybutanoic acid	PFMBA	0.999	7.07 × 10^−2^	0.4	0.4–125	2.4	96	102	0	0
Nonafluoro-3,6-dioxaheptanoic acid	NFDHA	0.999	8.48 × 103	0.4	0.4–125	2.9	103	97	2	2
9-Chlorohexadecafluoro-3-oxanonane-1-sulfonic acid	9Cl-PF3ONS	0.999	2.29 × 10^0^	0.8	0.8–250	2.3	102	100	3	3
11-Chloroeicosafluoro-3-oxaundecane-1-sulfonic acid	11Cl-PF3OUdS	0.999	1.43 × 10^0^	0.8	0.8–250	3.0	102	100	2	2
Perfluoro(2-ethoxyethane) sulfonic acid	PFEESA	0.999	2.53 × 10^−1^	0.4	0.4–125	1.5	100	101	1	1
3-Perfluoropropyl propanoic acid	3:3FTCA	0.999	2.76 × 10^−2^	1.0	1.0–312	3.0	98	100	0	0
2*H*,2*H*,3*H*,3*H*-Perfluorooctanoic acid	5:3FTCA	0.999	6.17 × 10^−2^	5.0	5.0–1560	2.4	101	100	0	0
3-Perfluoroheptyl propanoic acid	7:3FTCA	0.999	3.97 × 10^−2^	5.0	5.0–1560	3.2	101	99	1	1

**Table 2:** Quantitative results of the migration test for food simulant A (10% ethanol) and food simulant D1 (50% ethanol).

Compounds	Food Simulants	Concentration									
		P1		P2		P3		P4		P5	
		ng/g	ng/dm^2^	ng/g	ng/dm^2^	ng/g	ng/dm^2^	ng/g	ng/dm^2^	ng/g	ng/dm^2^
PFHxA	10% ethanol	152.7 ± 3.0	201.2 ± 0.3	13.2 ± 0.4	17.3 ± 0.7	23.7 ± 1.7	31.3 ± 0.6	32.7 ± 2.0	37.7 ± 0.2	14.9 ± 0.3	18.1 ± 0.3
	50% ethanol	166.6 ± 10.1	215.9 ± 9.1	14.1 ± 0.1	18.6 ± 0.7	18.5 ± 0.3	25.0 ± 0.4	48.7 ± 1.8	60.6 ± 4.6	17.3 ± 1.2	23.4 ± 1.3
PFHpA	10% ethanol	16.2 ± 0.6	21.3 ± 0.4	14.3 ± 0.7	18.7 ± 0.3	13.2 ± 0.7	17.5 ± 0.6	39.5 ± 2.4	45.8 ± 5.7	17.2 ± 1.4	20.7 ± 1.3
	50% ethanol	16.8 ± 0.8	21.8 ± 1.3	14.1 ± 0.7	18.6 ± 0.2	9.7 ± 0.6	13.1 ± 0.7	68.3 ± 0.9	85.0 ± 3.0	20.3 ± 0.6	27.4 ± 1.2
PFOA	10% ethanol	5.2 ± 0.4	6.9 ± 0.7	114.9 ± 4.6	150.3 ± 2.4	166.3 ± 8.1	220.1 ± 2.9	152.0 ± 5.4	175.6 ± 8.1	165.6 ± 11.9	199.5 ± 13.2
	50% ethanol	5.8 ± 0.2	7.5 ± 0.4	118.3 ± 6.5	156.5 ± 2.5	122.9 ± 3.0	166.4 ± 2.7	274.0 ± 7.0	340.9 ± 5.6	160.9 ± 4.3	216.9 ± 2.1
PFNA	10% ethanol	-	-	21.9 ± 1.5	28.7 ± 1.8	22.9 ± 0.9	30.4 ± 0.5	42.5 ± 2.8	49.2 ± 6.1	27.1 ± 0.7	32.6 ± 1.2
	50% ethanol	-	-	27.0 ± 0.8	35.8 ± 0.5	18.9 ± 1.2	25.6 ± 1.4	94.6 ± 1.9	117.7 ± 2.2	25.2 ± 0.5	33.9 ± 0.9
PFDA	10% ethanol	-	-	31.2 ± 1.9	40.8 ± 3.4	33.5 ± 2.5	44.2 ± 0.9	29.1 ± 1.1	33.6 ± 1.6	31.8 ± 3.6	38.4 ± 4.9
	50% ethanol	-	-	63.9 ± 4.1	84.5 ± 3.8	53.2 ± 1.9	72.1 ± 1.5	143.8 ± 7.4	178.9 ± 3.2	36.2 ± 2.6	48.7 ± 2.2
PFUnA	10% ethanol	-	-	-	-	-	-	-	-	-	-
	50% ethanol	-	-	8.6 ± 0.5	11.4 ± 0.4	10.2 ± 0.4	13.8 ± 0.7	26.5 ± 1.1	32.9 ± 0.2		
PFDoA	10% ethanol	-	-	-	-	-	-	-	-	-	-
	50% ethanol	-	-	24.1 ± 0.9	31.8 ± 0.2	22.4 ± 0.5	30.4 ± 0.5	51.7 ± 2.3	64.3 ± 0.8		

**Table 3:** The sum of PEQ based on the RPF in food simulant A (10% ethanol) and food simulant D1 (50% ethanol).

Compounds	RPF [34]	Food Simulants	PEQ (ng/g)				
			P1	P2	P3	P4	P5
PFHxA	0.01	10% ethanol	1.5	0.1	0.2	0.3	0.2
		50% ethanol	1.7	0.1	0.2	0.5	0.2
PFHpA	0.01 ≤ RPF ≤ 1	10% ethanol	0.2 ≤ PEQ ≤ 16.2	0.1 ≤ PEQ ≤ 14.3	0.1 ≤ PEQ ≤ 13.2	0.4 ≤ PEQ ≤ 39.5	0.2 ≤ PEQ ≤ 17.2
		50% ethanol	0.2 ≤ PEQ ≤ 16.8	0.1 ≤ PEQ ≤ 14.1	0.1 ≤ PEQ ≤ 9.7	0.7 ≤ PEQ ≤ 68.3	0.2 ≤ PEQ ≤ 20.3
PFOA	1	10% ethanol	5.2	114.9	166.3	152.0	165.6
		50% ethanol	5.8	118.3	122.9	274.0	160.9
PFNA	10	10% ethanol	-	219.4	229.7	424.6	270.7
		50% ethanol	-	270.3	188.8	945.7	251.8
PFDA	4 ≤ RPF ≤ 10	10% ethanol	-	124.7 ≤ PEQ ≤ 311.8	133.8 ≤ PEQ ≤ 334.6	116.3 ≤ PEQ ≤ 290.8	127.2 ≤ PEQ ≤ 318.0
		50% ethanol	-	255.4 ≤ PEQ ≤ 638.5	212.9 ≤ PEQ ≤ 532.3	575.2 ≤ PEQ ≤ 1437.9	144.6 ≤ PEQ ≤ 361.6
PFUnA	4	10% ethanol	-	-	-	-	-
		50% ethanol	-	34.4	40.8	105.9	-
PFDoA	3	10% ethanol	-	-	-	-	-
		50% ethanol	-	72.2	67.3	155.2	-
Sum PEQ (ng/g)		10% ethanol	6.9 ≤ PEQ ≤ 22.9	459.3 ≤ PEQ ≤ 660.6	530.2 ≤ PEQ ≤ 744.1	693.7 ≤ PEQ ≤ 907.3	563.9 ≤ PEQ ≤ 771.7
		50% ethanol	7.6 ≤ PEQ ≤ 24.3	750.9 ≤ PEQ ≤ 1147.9	632.9 ≤ PEQ ≤ 961.9	2057.1 ≤ PEQ ≤ 2987.5	557.7 ≤ PEQ ≤ 794.8

**Table 4:** Dietary exposure and risk assessment results for food simulant A (10% ethanol) and food simulant D1 (50% ethanol).

Approach	Dietary Exposure and Risk Assessment	Food Simulant	Samples				
			P1	P2	P3	P4	P5
EFSA approach (TDI: 0.63 ng/kg bw/day)	Sum of PFOA and PFNA (ng/g)	10% ethanol	5.2	136.9	189.3	194.5	192.7
		50% ethanol	5.8	145.3	141.8	370.4	186.1
	CEDI ^a^	10% ethanol	0.01	0.2	0.3	0.3	0.3
		50% ethanol	0.01	0.2	0.2	0.5	0.3
	HR ^b^	10% ethanol	0.01	0.3	0.4	0.4	0.4
		50% ethanol	0.01	0.3	0.3	0.8	0.4
RPF approach (RfD of PFOA: 0.03 ng/kg bw/day)	Sum of PEQ (ng/g)	10% ethanol	6.9 ≤ PEQ ≤ 22.9	459.3 ≤ PEQ ≤ 660.6	530.2 ≤ PEQ ≤ 744.1	693.7 ≤ PEQ ≤ 907.3	563.9 ≤ PEQ ≤ 771.7
		50% ethanol	7.6 ≤ PEQ ≤ 24.3	750.9 ≤ PEQ ≤ 1147.9	632.9 ≤ PEQ ≤ 961.9	2057.1 ≤ PEQ ≤ 2987.5	557.7 ≤ PEQ ≤ 794.8
	CEDI ^a^	10% ethanol	0.01 ≤ CEDI ≤ 0.03	0.7 ≤ CEDI ≤ 0.9	0.8 ≤ CEDI ≤ 1.1	1.0 ≤ CEDI ≤ 1.3	0.8 ≤ CEDI ≤ 1.1
		50% ethanol	0.01 ≤ CEDI ≤ 0.03	1.1 ≤ CEDI ≤ 1.6	0.9 ≤ CEDI ≤ 1.4	2.9 ≤ CEDI ≤ 4.3	0.8 ≤ CEDI ≤ 1.1
	HR ^b^	10% ethanol	0.3 ≤ HR ≤ 1.1	21.9 ≤ HR ≤ 31.5	25.2 ≤ HR ≤ 35.4	33.0 ≤ HR ≤ 43.2	26.9 ≤ HR ≤ 36.7
		50% ethanol	0.4 ≤ HR ≤ 1.2	35.8 ≤ HR ≤ 54.7	30.1 ≤ HR ≤ 45.8	98.0 ≤ HR ≤ 142.3	26.6 ≤ HR ≤ 37.8

^a^ CEDI (cumulative estimated daily intake) values were calculated according to US FDA guidance, where CEDI = the sum of PFOA and PFNA (ng/g) or the sum of PEQ, multiplied by CF (Microwave susceptor = 0.001), f_T_ (since the US FDA does not provide specific f_T_ values for microwave susceptor materials, and the microwave popcorn bag samples used in this study come into contact exclusively with popcorn, which is classified as a fatty food, an f_T_ value of 1 was assumed), and the amount consumed per day (one bag per day, 100 g), divided by bw (70 kg). ^b^ The HR value is the ratio of the calculated CEDI to the EFSA’s TDI for the combined total of PFOA, PFNA, PFHxS, and PFOS (0.63 ng/kg bw/day) or EPA’s RfD for PFOA (0.03 ng/kg bw/day).

**Table 5:** Compounds analyzed using UHPLC-Orbitrap MS, including their RT, ions detected, precursor ions and quantification reference compound.

Abbreviation	RT (min)	Ions Detected	Precursor Ion (*m*/*z*)	Quantification Reference Compound
PFBA	4.1	[M − H]^−^	212.9792	^13^C_4_-PFBA
PFPeA	5.4	[M − H]^−^	262.9760	^13^C_5_-PFPeA
PFHxA	6.1	[M − H]^−^	312.9728	^13^C_5_-PFHxA
PFHpA	6.7	[M − H]^−^	362.9696	^13^C_4_-PFHpA
PFOA	7.1	[M − H]^−^	412.9664	^13^C_8_-PFOA
PFNA	7.5	[M − H]^−^	462.9632	^13^C_9_-PFNA
PFDA	7.9	[M − H]^−^	512.9600	^13^C_6_-PFDA
PFUnA	8.3	[M − H]^−^	562.9568	^13^C_7_-PFUnA
PFDoA	8.7	[M − H]^−^	612.9537	^13^C_2_-PFDoA
PFTrDA	9.0	[M − H]^−^	662.9505	^13^C_2_-PFTeDA
PFTeDA	9.4	[M − H]^−^	712.9473	^13^C_2_-PFTeDA
PFBS	6.1	[M − H]^−^	298.9430	^13^C_3_-PFBS
PFPeS	6.7	[M − H]^−^	348.9398	^13^C_3_-PFHxS
PFHxS	7.2	[M − H]^−^	398.9366	^13^C_3_-PFHxS
PFHpS	7.6	[M − H]^−^	448.9334	^13^C_8_-PFOS
PFOS	8.0	[M − H]^−^	498.9302	^13^C_8_-PFOS
PFNS	8.4	[M − H]^−^	548.9270	^13^C_8_-PFOS
PFDS	8.8	[M − H]^−^	598.9238	^13^C_8_-PFOS
PFDoS	9.5	[M − H]^−^	698.9174	^13^C_8_-PFOS
4:2FTS	5.9	[M − H]^−^	326.9743	^13^C_2_-4:2FTS
6:2FTS	6.9	[M − H]^−^	426.9679	^13^C_2_-6:2FTS
8:2FTS	7.7	[M − H]^−^	526.9615	^13^C_2_-8:2FTS
PFOSA	10.8	[M − H]^−^	497.9462	^13^C_8_-PFOSA
NMeFOSA	12.3	[M − H]^−^	511.9619	D_3_-NMeFOSA
NEtFOSA	12.7	[M − H]^−^	525.9775	D_5_-NEtFOSA
NMeFOSAA	8.2	[M − H]^−^	569.9673	D_3_-NMeFOSAA
NEtFOSAA	8.4	[M − H]^−^	583.9830	D_5_-N-EtFOSAA
NMeFOSE	12.2	[M + CH_3_COO]^−^	616.0092	D_7_-NMeFOSE
NEtFOSE	12.5	[M + CH_3_COO]^−^	630.0248	D_9_-NEtFOSE
HFPO-DA	6.3	[M − H − CO_2_]^−^	284.9784	_13_C_3_-HFPO-DA
ADONA	6.8	[M − H]^−^	376.9689	_13_C_3_-HFPO-DA
PFMPA	4.8	[M − H]^−^	228.9741	_13_C_5_-PFPeA
PFMBA	5.7	[M − H]^−^	278.9709	_13_C_5_-PFPeA
NFDHA	6.1	[M − H]^−^	294.9658	_13_C_5_-PFHxA
9Cl-PF3ONS	8.4	[M − H]^−^	530.8956	_13_C_3_-HFPO-DA
11Cl-PF3OUdS	9.1	[M − H]^−^	630.8892	_13_C_3_-HFPO-DA
PFEESA	6.4	[M − H]^−^	314.9379	_13_C_5_-PFHxA
3:3FTCA	5.3	[M − H]^−^	241.0105	_13_C_5_-PFPeA
5:3FTCA	6.9	[M − H]^−^	341.0041	_13_C_5_-PFHxA
7:3FTCA	8.2	[M − H]^−^	440.9977	_13_C_5_-PFHxA

## Data Availability

The data presented in this study are available in the article.

## Abbreviations

The following abbreviations are used in this manuscript:

PFASs	Perfluoroalkyl and polyfluoroalkyl substances.
FCMs	Food contact materials.
HRMS	High-resolution mass spectrometry.
EFSA	European Food Safety Authority.
TWI	Tolerable weekly intake.
bw	Body weight.
IARC	International Agency for Research on Cancer.
HR	Hazard ratio.
R	Coefficient of correlation.
LOQ	Limit of quantification.
RSE	Relative standard error.
TFDA	Taiwan Food and Drug Administration.
RPF	Relative potency factor.
PEQ	Perfluorooctanoic acid equivalents.
CEDI	Cumulative estimated daily intake.
EWI	Estimated weekly intake.
QqQ	Triple quadrupole.
LC	Liquid chromatographic separation.
ESI	Electrospray ionization.
RT	Retention time.
S/N	Signal-to-noise.
CF	Consumption factors.
f_T_	Food-type distribution factors.
RfD	Reference dose.
